# Trends and associated factors of HIV, HCV and syphilis infection among different drug users in the China–Vietnam border area: an 11-year cross-sectional study (2010–2020)

**DOI:** 10.1186/s12879-023-08239-3

**Published:** 2023-09-04

**Authors:** Tong Luo, Zhaosen Lin, Zhenxian Wu, Ping Cen, Aidan Nong, Rongye Huang, Jianhua Che, Fengfeng Liang, Yuan Yang, Jie Liu, Li Huang, Jie Cai, Yanyun Ou, Li Ye, Lijuan Bao, Bingyu Liang, Hao Liang

**Affiliations:** 1 Guangxi Key Laboratory of AIDS Prevention and Treatment, School of Public Health, Guangxi Medical University, Nanning, 530021 Guangxi China; 2 Collaborative Innovation Centre of Regenerative Medicine and Medical Bioresource Development and Application Co-constructed by the Province and Ministry, Life Science Institute, Guangxi Medical University, Nanning, 530021 Guangxi China; 3 Qinzhou Center for Disease Control and Prevention, Qinzhou, 535000 Guangxi China; 4 Chongzuo Center for Disease Control and Prevention, Chongzuo, 532200 Guangxi China

**Keywords:** HIV, HCV, Syphilis, China–Vietnam border, Drug users

## Abstract

**Background:**

Data on recent human immunodeficiency virus (HIV), hepatitis C virus (HCV) and syphilis prevalence among drug users in the Southwest China are sparse despite the high burden of drug use. This study aims at assessing the prevalence trends and related factors of HIV, HCV and syphilis infection among different drug users in the China–Vietnam border area.

**Methods:**

A continuous cross-sectional survey was conducted among drug users from 2010 to 2020 in the China–Vietnam border area. Chi-square trend tests were used to assess the trend of HIV, HCV and syphilis prevalence and the proportion for drug type used by drug users. Multivariate logistic regression was used to identify associated factors of HIV, HCV and syphilis infection in different drug users.

**Results:**

In this study, a total of 28,951 drug users were included, of which 27,893 (96.45%) male, 15,660 (54.09%) aged 13–34 years, 24,543 (84.77%) heroin-only users, 2062 (7.12%) synthetic drug-only (SD-only) users and 2346 (8.10%) poly-drug users. From 2010 to 2020, the proportion of heroin-only users decreased from 87.79% to 75.46%, whereas SD-only users and poly-drug users increased from 5.16% to 16.03%, and from 7.05% to 8.52%, respectively. The prevalence of HIV, HCV, and syphilis during the study period declined from 12.76%, 60.37% and 5.72% to 4.35%, 53.29% and 4.53%, respectively, among heroin-only users and declined from 18.30%, 66.67% and 15.69% to 6.95%, 27.81% and 5.35%, respectively, among poly-drug users; however, the prevalence of HIV and HCV among SD-only users increased from 0.89% and 8.93% to 2.84% and 18.75%, respectively. Having ever injected drugs and needle sharing were common associated factors for both HIV and HCV infection among poly-drug users and heroin-only users. Aged ≥ 35 years old was an associated factor for HIV, HCV and syphilis infection among the SD-only users. Female drug users were at high risk of contracting syphilis among three different drug users.

**Conclusions:**

The prevalence of HIV, HCV and syphilis among heroin-only users and poly-drug users decreased during the study period. However, the prevalence of HIV and HCV among SD-only users increased. Comprehensive intervention strategies, particularly focusing on the SD-only users are needed in order to bring down the disease burden in this population in the China–Vietnam border areas.

**Supplementary Information:**

The online version contains supplementary material available at 10.1186/s12879-023-08239-3.

## Background

In 2019, approximately 275 million people worldwide used non-prescribed drugs and approximately 36 million suffered from drug use symptoms [1]. Drug use not only causes physical harm to drug users, but also increases the risk of HIV (human immunodeficiency virus) and HCV (hepatitis C virus) transmission [2]. Although, in China, the main mode of HIV transmission changed from drug use to sexual transmission in 2005 [3], drug use is still an important mode of HIV transmission. In particular, injecting drugs and engaging in unprotected high-risk sexual activities after drug use are common practice [3–6]. According to the United Nations Office on Drugs and Crime (UNODC), about one in eight people who inject drugs worldwide are living with HIV [1] and numerous studies have shown that injecting drugs is an important associated factor for HCV infection [1]. The World Health Organization (WHO) estimates that nearly a quarter (23%) of the 1.7 million new HCV infections worldwide in 2015 were due to drug injection [7]. Syphilis was epidemiologically closely related to AIDS infection [8, 9]and the incidence of HIV has been reported to be as high as 20% in the decade following a syphilis diagnosis [10].

Although China implements a strong anti-drug policy, it also conforms to the shifting global patterns of drug use [11, 12]. By the end of 2020, there were 1.801 million drug addicts in China: a decrease for the third consecutive year [13]. In 2000, heroin users in China accounted for 96.8% whereas synthetic drug users accounted for only 1.1% of all drug users [14]. However, in 2020, among the 1.801 million existing drug addicts, 1.031 million abused synthetic drugs, accounting for 57.2% of the total, and 734,000 abused heroin, accounting for 40.8% of the total [13]. Changes in drug use patterns and the gradual increase of synthetic and poly-drug users have created more favorable conditions for the spread of blood-borne diseases [12]. Because synthetic drugs are often used recreationally in clubs, they are also known as club drugs [15]. Their effects on the nervous system are very rapid, and because of their strong influence on sexual behavior they often increase the incidence of high-risk sexual behaviors [3, 16]. Existing studies have shown that a high prevalence of having multiple sexual partners and unprotected sex after the use of synthetic drugs is associated with the promotion of HIV and HCV infection [17, 18]. In addition, females and lower education were associated with syphilis infection among synthetic drug users [19].

Guangxi is located in the southwest of China, bordering Vietnam. It is the closest to the “Golden Triangle” except for Yunnan, and it is also the only autonomous region in the southwest with border and coastal cities. The geographical convenience makes Guangxi one of the important passages for “Golden Triangle” drugs to pass through Vietnam and flow into China. With the intensified crackdown on the China–Vietnam drug-trafficking channel, drug criminals have turned their attention to the China–Vietnam border. In recent years, the problem of drug smuggling in the China-Vietnam border area of Guangxi has continued to escalate and has been identified by the Ministry of Public Security as the second largest entry channel for drug smuggling in China after Yunnan [20]. Although different studies of drug users in China–Vietnam have been conducted previously, there is a lack of recent data on the temporal changes in HIV, HCV and syphilis prevalence and the associated factors for HIV, HCV and syphilis infection among different drug users. This study recruited drug users from the China–Vietnam border area from 2010 to 2020, assessing the prevalence trends and related factors of HIV, HCV and syphilis among different drug users.

## Methods

### Study setting

From 2010 to 2020, a continuous cross-sectional survey, as part of the routine sentinel drug user surveillance, was conducted among the drug users in the China–Vietnam border area of Guangxi from March to August every year.

### Study participants and procedures

**Fig. 1:**
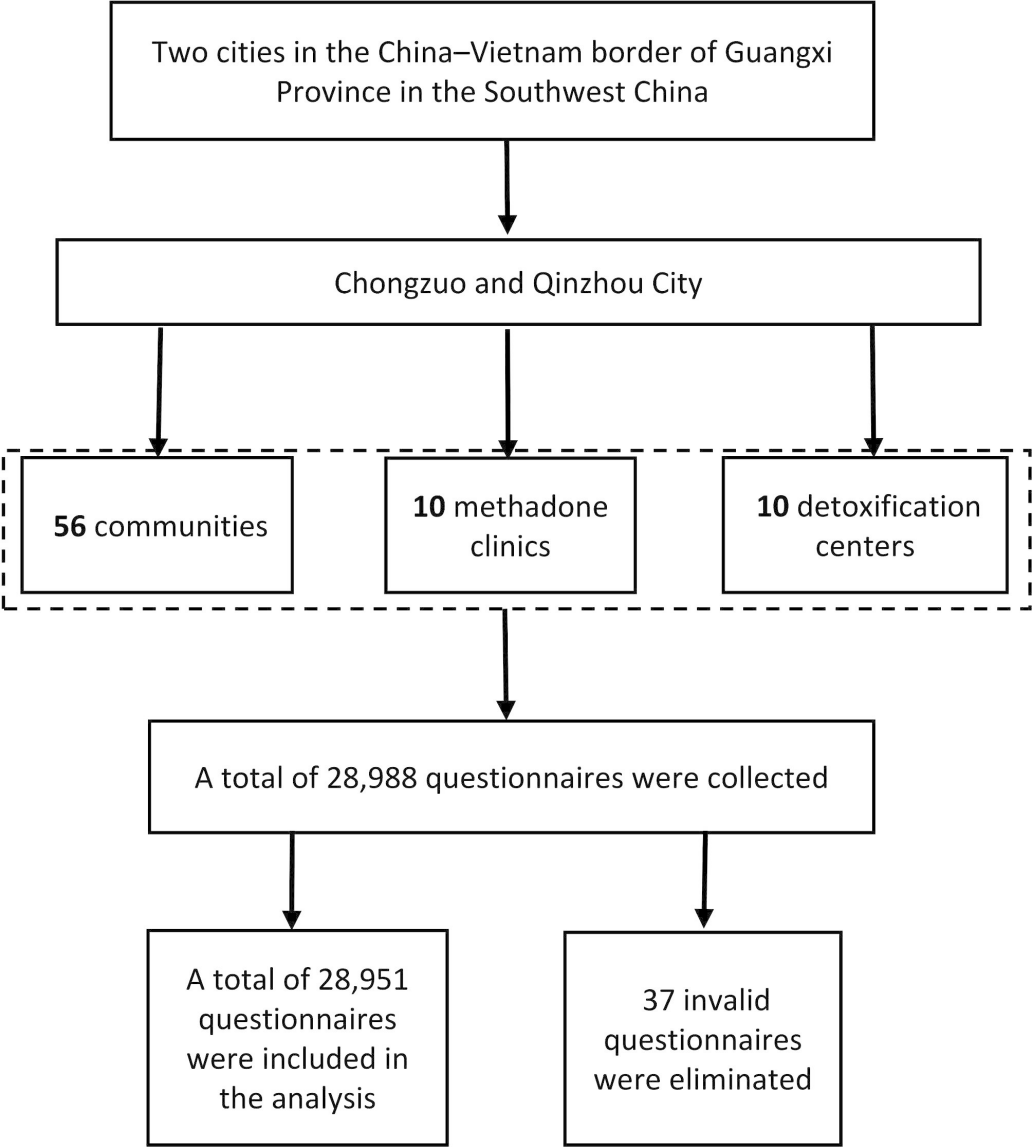
Flow chart of participants recruitment. Participants include: (1) Drug users recruited by peer educators through snowball sampling. (2) Drug users in the detoxification centers and methadone clinics during the recruitment period. Invalid questionnaires include: (1) without demographic data (2) without drug abuse records

Study participants were recruited from various detoxification centers, methadone clinics, and communities using a combination of non-probability sampling techniques: convenience sampling and respondent-driven sampling (Fig. 1). The specific practices are as follows: (1) Some participants were recruited through community-based outreach from communities, and outreach workers from Chongzuo City, Qinzhou City and other district Centers for Disease Control and Prevention (CDC) distributed recruitment information about the surveys throughout the local communities and directly contacted known drug users. (2) Part of participants were recruited through responded sampling by peer educators and by referral from participants who were already enrolled. (3) Drug users who have been assessed by designated medical institutions for drug use disorders and forced by the police to enter the detoxification centers. (4) Drug users from methadone clinics.

Drug users who met the eligibility criteria in these places were recruited into the survey. Eligibility criteria included the following: (1) aged at least 13 years; (2) reported using at least one type of illicit drug (inhalation, oral administration or injection) in the past three months; (3) provision of written informed consent. Exclusion criteria included: (1) aged younger than 13 years; (2) no history of drug use; (3) unwilling to provide informed consent.

Before the survey, participants were informed about the purpose of the survey and the need to take blood samples to test for HIV, HCV and syphilis. After obtaining written informed consent from participants, trained CDC staff conducted face-to-face interviews in a private room using a structured questionnaire.

A unique identification number was assigned to eliminate duplicate registrations and ensure confidentiality. During the survey, interviewers provided explanations and instructions to help participants understand any questions in the questionnaire.

After the questionnaire survey, trained specialized staff collected the participants` blood samples in order to undertake serological tests for HIV, HCV and syphilis prevalence. The local Center for Disease Control and Prevention conducts laboratory tests for HIV, HCV and syphilis. Participants who test positive for HIV, HCV or syphilis in the laboratory were consulted and referred to the local treatment center for treatment.

### Socio-demographic, behavioural, and AIDS related knowledge variables

The survey collected socio-demographic data including age, marital status, ethnicity and years of education. In this study, synthetic drug includes methamphetamine, demerol, ketamine, ecstasy, magu, diazepam and Gamma-Hydroxybutyrate. The participants were divided into three categories according to the kind of drugs they used: (1) heroin-only users: those who have never used synthetic drugs before the survey; (2) synthetic drug-only (SD-only) users: those who have never used heroin before the survey; (3) poly-drug users: those who had used both heroin and at least one synthetic drugs (includes methamphetamine, demerol, ketamine, ecstasy, magu, diazepam and Gamma-Hydroxybutyrate) in last three months.

HIV/AIDS related knowledge was assessed with an 8-question [21], six or more correct answers are considered as aware. This assessment criteria is widely used in China [22, 23].

Behavioral variables were measured by questions including whether they had ever injected drugs, shared needles with others, had sex following drug consumption, engaged in commercial sex, received free condom or HIV testing and counseling services, peer education, free methadone maintenance therapy or clean needles.

### Ethics statement

Each participant was compensated 20 RMB (about $3 USD) for participating in this study, which was approved by the Human Research Ethics Committee of Guangxi Medical University (Ethical Review No. 2013 − 130).

### Laboratory screening

Serological detection of HIV, HCV and syphilis was based on the national diagnostic guidelines in China [24–26]. Western blotting (WB) validation (HIV Blot 2.2 WB; Genelabs Diagnostic, Singapore) were used to diagnose HIV only after positive samples are screened by enzyme-linked immunosorbent assay (ELISA) (Wantai Biological Pharmaceutical Co., Beijing, China). For HCV, samples were tested for HCV antibodies by repeated independent ELISA testing (Wantai Biological Pharmaceutical Co., Beijing, China) [27]. For syphilis, rapid plasma regain (RPR) (Rongsheng Biotechnical Company, Shanghai, China) was used as a screening test and positive samples were confirmed by Treponema pallidum particle agglutination (TPPA) (Serodia; Fujirebio, Fuji, Tapan) or toluidine red unheated serum test (TRUST) (Rongsheng Biotechnical Company, Shanghai China).

### Statistical analysis

The collected data were double entered with EpiData3.0. For sociodemographic characteristics, the proportions were calculated for the three groups: poly-drug users, heroin-only users and SD-only users. All analyses were based on SPSS version 26.0 (SPSS, Inc, Chicago, IL, USA). A chi-square test was used to evaluate key factors distinguishing heroin-only users, SD-only users and poly-drug users. A chi-square trend test was used to test the significance of the time trend. Univariate logistic regression analyses were performed for each independent variable of uptake of HIV, HCV and syphilis infection, with crude odds ratios (ORs) and 95% confidence intervals (CIs) indicated. Variables with a significant univariate level (*p* < 0.2) were input into the multivariate logistic regression model to calculate the adjusted ORs (AORS) and 95% CIs against the dependent variable for each group: *p* < 0.05 was considered significant in the final logistic regression models. All confidence intervals (CIs) proposed were 95% CIs and all *p* values were two-sided *p* values.

## Results

### Socio-demographic characteristics

A total of 28,951 interviewers participated in this study. There were 24,543 (84.77%) heroin-only users,2062 (7.12%) SD-only users and 2346 (8.10%) poly-drug users among the survey respondents, categorized according to type of self-reported drug use. As shown in Table 1, most of the participants were male (27,893; 96.35%), aged 13–34 years (15,660; 54.09%), “other” ethnic groups (16,017; 55.32%), divorced/widowed/unmarried (17,732; 61.25%) and time spent in education < 9 years (26,981; 93.20%). Also, most of the respondents (27,326; 94.39%) had some HIV-related knowledge. The proportion of males was highest among heroin-only users (23,746; 96.75%) and lowest among SD-only users (1895; 91.90%; *p* < 0.01). The SD-only users were the youngest (1442; 69.93%) and best educated (242; 11.74%), the poly-drug users were the oldest (1214; 51.75%) and the heroin-only users were the least educated (1503; 6.12%; *p* < 0.01).

**Table 1:** Demographic characteristics of heroin-only users, SD-only users, and poly-drug users

Variables	Total n (%)	Heroin-only n (%)	Synthetic drug-only n (%)	Poly-drug n (%)	*χ²*	*P*
**Gender**					128.064	< 0.001
Male	27,893(96.35)	23,746(96.75)	1895(91.90)	2252(95.99)		
Female	1058(3.65)	797(3.25)	167(8.10)	94(4.01)		
**Age(years)**					246.472	< 0.001
13–34	15,660(54.09)	13,086(53.32)	1442(69.93)	1132(48.25)		
≥ 35	13,291(45.91)	11,457(46.68)	620(30.07)	1214(51.75)		
**Marital Status**					26.525	< 0.001
Married/Cohabitated	11,219(38.75)	9644(39.29)	778(37.73)	797(33.97)		
Divorced/Widowed/ Unmarried	17,732(61.25)	14,899(60.71)	1284(62.27)	1549(66.03)		
**Education(years)**					125.727	< 0.001
< 9	26,981(93.20)	23,040(93.88)	1820(88.26)	2121(90.41)		
≥ 9	1970(6.80)	1503(6.12)	242(11.74)	225(9.59)		
**Ethnicity**					19.439	< 0.001
Han	12,934(44.68)	10,831(44.13)	979(47.48)	1124(47.91)		
Other	16,017(55.32)	13,712(55.87)	1083(52.52)	1222(52.09)		
**Awareness of HIV-related knowledge**					15.363	< 0.001
Yes	27,326(94.39)	23,210(94.57)	1943(94.23)	2173(92.63)		
No	1625(5.61)	1333(5.43)	119(5.77)	173(7.37)		

### Behavioral characteristics

As shown in Table 2, among the three groups the poly-drug users had a higher proportion (1945; 82.91%) of those who had ever injected drugs than the SD-only users (324; 15.71%) and heroin-only users (19,182; 78.16%; *p* < 0.01). The proportion self-reporting needle sharing with others was significantly lower in the SD-only users (98; 4.75%) than among the heroin-only users (6627; 27.00%) and poly-drug users (632; 26.94; *p* < 0.01). In contrast, SD-only users (224; 20.42%) reported the highest rate of sexual activities following drug consumption compared with the heroin-only users (932; 9.85%) and poly-drug users (173; 16.96%; *p* < 0.01). The poly-drug users were more likely (589; 32.27%) to have ever engaged in commercial sex than the heroin-only users (4751; 24.11%) and SD-only users (487; 29.88%; *p* < 0.01). Most drug users received HIV-related intervention services. However, compared with heroin-only users and poly-drug users, the proportion of SD-only users who had received intervention services was the lowest (*p* < 0.01) because merely 67.41% (1390) had ever received free condoms or HIV testing and counseling services. The proportions of SD-only users who had ever received methadone maintenance therapy and clean-needle/peer education services were only 17.51% (361) and 16.93% (349), respectively.

**Table 2:** Behavioral characteristics and prevention services of heroin-only, SD-only and poly-drug users

Variables	Total n (%)	Heroin-only n (%)	Synthetic drug-only n (%)	Poly-drug n (%)	*χ²*	*P*
**Having ever injected drugs**					3967.412	< 0.001
Yes	21,451(74.09)	19,182(78.16)	324(15.71)	1945(82.91)		
No	7500(25.91)	5361(21.84)	1738(84.29)	401(17.09)		
**Needle sharing**					499.923	< 0.001
Yes	7357(25.41)	6627(27.00)	98(4.75)	632(26.94)		
No	21,594(74.59)	17,916(73.00)	1964(95.25)	1714(73.06)		
**Having sex following drug consumption**					141.226	< 0.001
Yes	1329(11.48)	932(9.85)	224(20.42)	173(16.96)		
No	10,251(88.52)	8531(90.15)	873(79.58)	847(83.04)		
**Having ever engaged in commercial sex**					79.838	< 0.001
Yes	5827(25.16)	4751(24.11)	487(29.88)	589(32.27)		
No	17,333(74.84)	14,954(75.89)	1143(70.12)	1236(67.73)		
**Having ever received free condom or HIV testing and counseling services**					308.768	< 0.001
Yes	23,296(80.47)	20,146(82.08)	1390(67.41)	1760(75.02)		
No	5655(19.53)	4397(17.92)	672(32.59)	586(24.98)		
**Having ever received free methadone maintenance therapy or clean needles**					1993.844	< 0.001
Yes	18,299(63.21)	16,380(66.74)	361(17.51)	1558(66.41)		
No	10,652(36.79)	8163(33.26)	1701(82.49)	788(33.59)		
**Having ever received peer education services**					720.452	< 0.001
Yes	13,093(45.22)	11,670(47.55)	349(16.93)	1074(45.78)		
No	15,858(54.78)	12,873(52.45)	1713(83.07)	1272(54.22)		

### Trends of drug user proportion and HIV, HCV and syphilis prevalence

**Fig. 2:**
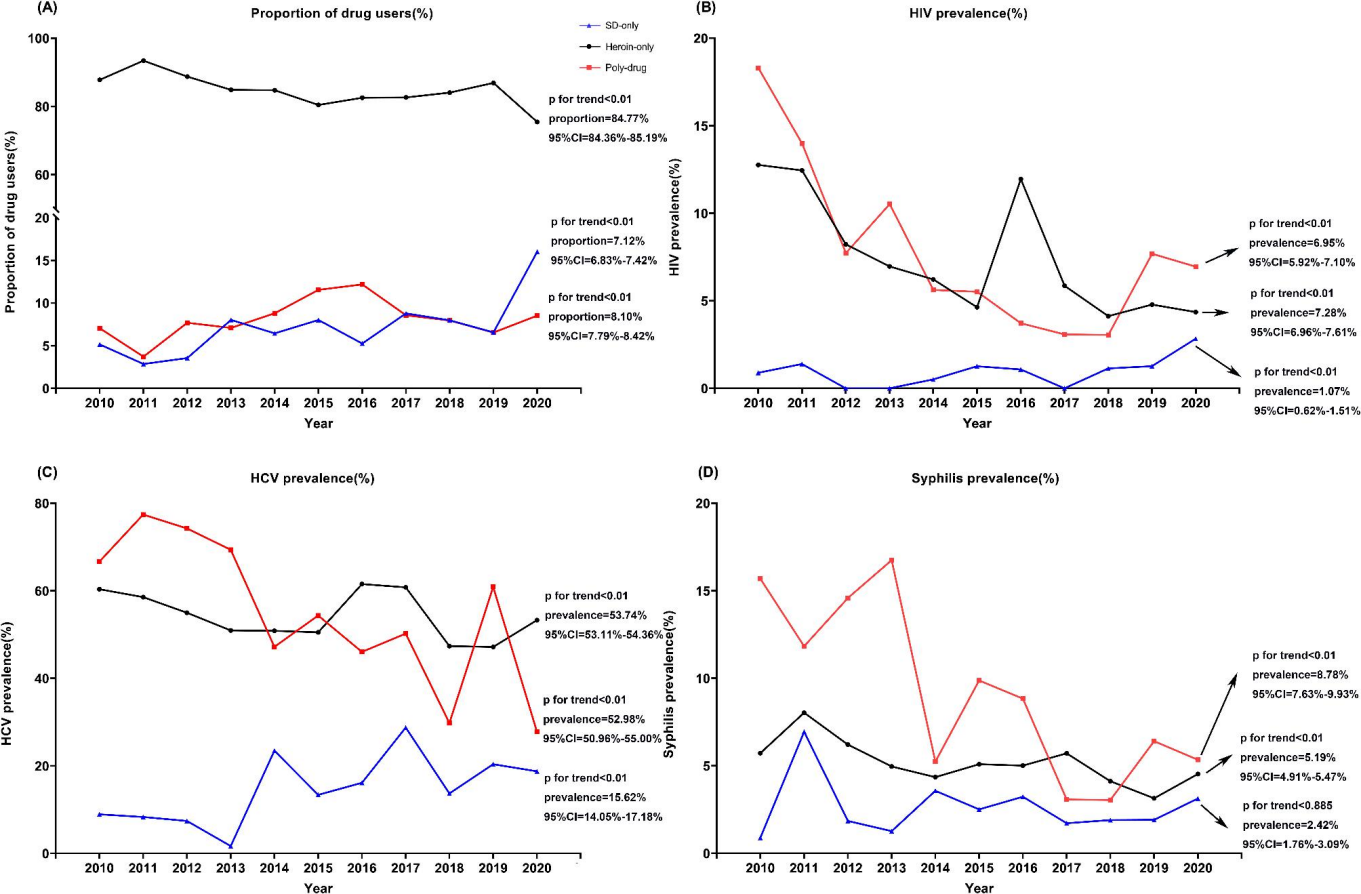
Trends of drug user proportion and HIV, HCV and syphilis prevalence of heroin-only users, SD-only users and poly-drug users during 2010–2020. (A) trends of proportion different drug users; (B) trends of HIV prevalence; (C) trends of HCV prevalence of;(D) trends of syphilis prevalence. Different colors represent results of different drug users. More information shown in the Supplementary Material Tables S1 and S2

As shown in Fig. 2, between 2010 and 2020 the proportion of heroin-only users among the drug users decreased from 87.79% (95%CI = 86.41-89.17%) to 75.46% (95%CI = 73.65-77.26%), while the proportions of SD-only and poly-drug users increased from 5.16% (95%CI = 4.23-6.09%) to 16.03% (95%CI = 14.49-17.56%) and from 7.05% (95%CI = 5.97-8.13%) to 8.52% (95%CI = 7.35-9.68%), respectively (Fig. 2A). HIV prevalence decreased significantly from 12.76% (95%CI = 11.26-14.26%) in 2010 to 4.35% (95%CI = 3.36-5.33%) in 2020 among heroin-only users, observably decreased from 18.30% (95%CI = 12.10-24.50%) to 6.95% (95%CI = 3.27-10.63%) among poly-drug users but increased from 0.89% (95%CI= 0.88-2.66%) to 2.84% (95%CI = 1.10-4.58%) among SD-only users (Fig. 2B). From 2010 to 2020, the prevalence of HCV and syphilis declined in both heroin-only and poly-drug users, with the prevalence of poly-drug users’ syphilis decreasing from 15.69% (95%CI = 9.86-21.51%) to 5.35% (95%CI = 2.09-8.60%) (Fig. 2C and D). However, the prevalence of HCV among SD-only users was on the rise: from 8.93% (95%CI = 3.57-14.29%) in 2010 to 18.75% (95%CI = 14.65-22.85%) in 2020. And between 2010 and 2020, among three diseases patients, the proportion of aged ≥ 35 years old increased. Detailed data are provided in Supplementary Material Tables S1-S3.

### Trends of drug users’ behavioral characteristics

Between 2010 and 2020, the proportion of having ever injected drugs among drug users decreased from 79.35% (95%CI = 77.65-81.06%) to 70.99% (95%CI = 67.52-71.37%), while the proportions of having ever engaged in commercial sex increased from 23.70% (95%CI = 21.90-25.49%) to 32.56% (95%CI = 29.63-35.48%). Between 2016 and 2020, the proportion of having sex following drug consumption decreased from 13.88% (95%CI = 11.80-15.97%) to 9.38% 95%CI = 8.16-10.60%). Detailed data are provided in Supplementary Material Tables S4.

### Factors associated with HIV, HCV and syphilis infections

**Fig. 3:**
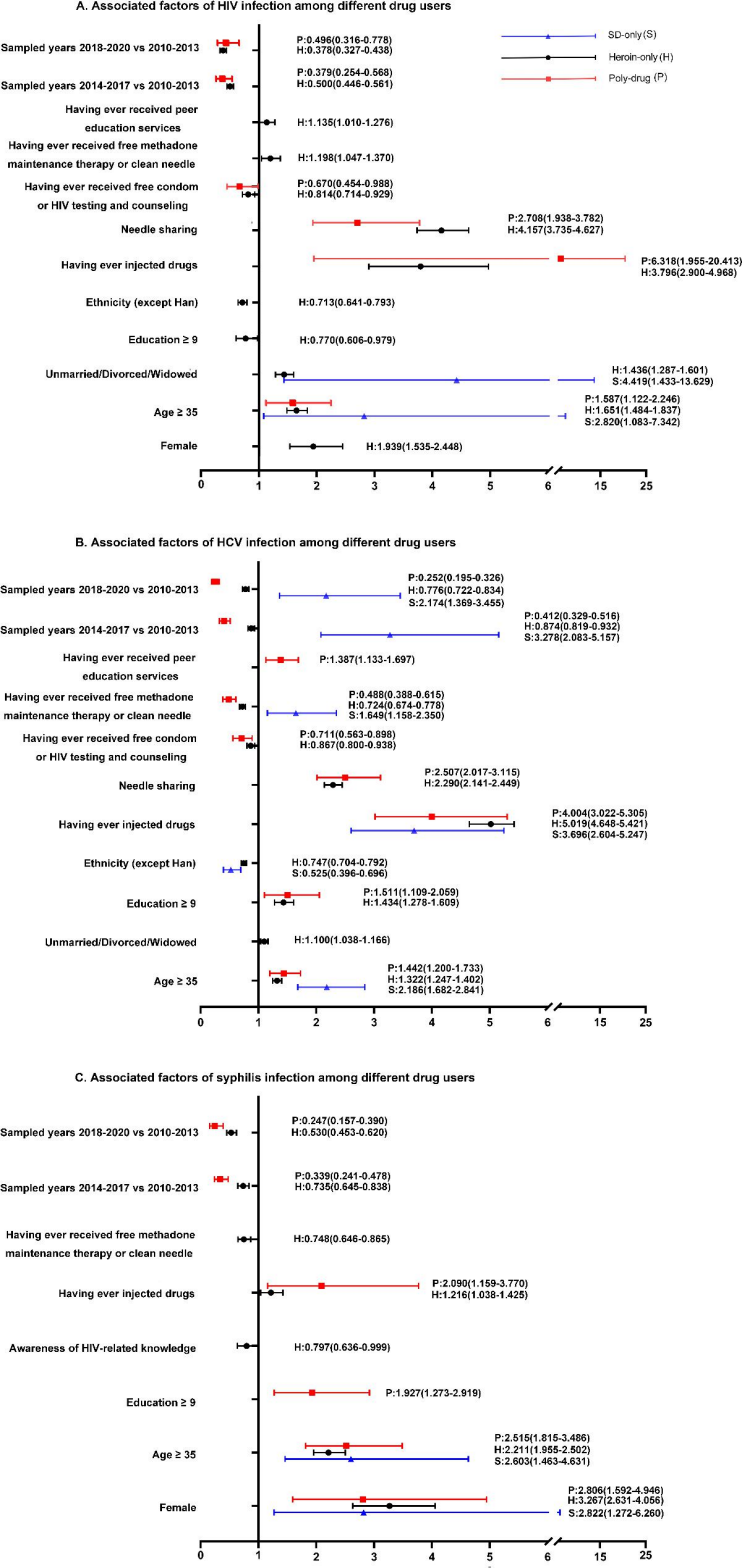
Associated factors of HIV, HCV and syphilis infection among different drug users. (A)Associated factors of HIV infection (B)Associated factors of HCV infection (C)Associated factors of syphilis infection. Different colors represent results of different drug users. More information in the Supplementary Material Tables S5–S7.

Having ever injected drugs and needle sharing were major associated factors for HIV infection among both heroin-only and poly-drug users (heroin-only: AOR = 3.796 and 4,157, 95%CI = 2.900–4.968 and 3.735–4.627; poly-drug: AOR = 6.318 and 2.708, 95%CI = 1.955–20.413 and 1.938–3.782, respectively, Fig. 3A). Aged ≥ 35 years (AOR = 2.820; 95%CI = 1.083–7.342) and unmarried/divorced/widowed marital status (AOR = 4.419; 95%CI = 1.433–13.629) were independently associated with HIV infection among SD-only users. Notably, women among the heroin-only users were more likely to be infected with HIV (AOR = 1.939; 95%CI = 1.535–2.448) than men. Having ever received free condoms or HIV testing and counseling services was a protective factor against HIV infection for both heroin-only users (AOR = 0.814; 95%CI = 0.714–0,929) and poly-drug users (AOR = 0.670; 95%CI = 0.454–0.988).

SD-only users aged ≥ 35 years (AOR = 2.186; 95%CI = 1.682–2.841) and those who had ever injected drugs (AOR = 3.696; 95%CI = 2.604–5.247) were more likely to be infected by HCV(Fig. 3B). In addition, with heroin-only and poly-drug users, having ever injected drugs (heroin-only: AOR = 5.019, 95%CI = 4.648–5.421; poly-drug: AOR = 4.004, 95%CI = 3.022–5.305) and needle sharing (heroin-only: AOR = 2.290, 95%CI = 2.141–2.449; poly-drug: AOR = 2.507, 95%CI = 2.017–3.115) were associated factors for HCV infection. Common protective factors for HCV infection among both heroin-only and poly-drug users were having ever received free condoms or HIV testing and counseling services (heroin-only: AOR = 0.867, 95%CI = 0.800–0.938; poly-drug: AOR = 0.711, 95%CI = 0.563–0.898) and having ever received free methadone maintenance therapy or clean needles (heroin-only: AOR = 0.724, 95%CI = 0.674–0.778; poly-drug: AOR = 0.488, 95%CI = 0.388–0.615).

Drug users aged ≥ 35 years and female were at greater risk of contracting syphilis among all three groups (Fig. 3C). Having ever injected drugs was not only associated with HIV and HCV infection but were also associated factors for syphilis infection among poly-drug users (AOR = 2.090 and 1.278, 95%CI = 1.159–3.770). Furthermore, receiving education for more than 9 years (AOR = 1.927; 95%CI = 1.273–2.919) was also associated with syphilis infection among poly-drug users. Notably, for heroin-only and poly-drug users, the risk of HIV, HCV and syphilis infection was reduced in the sampled year of 2014–2017 and 2018–2020 compared to the sampled year of 2010–2013. However, for SD-only users, there was an increase in HCV infection during the sampled year of 2014–2017 and 2018–2020 compared with the sampled year of 2010–2013. Detailed data are provided in Supplementary Material Tables S5–S7.

## Discussion

To our knowledge, in this work the survey period is the longest and the sample size (28,951) the largest in China, with 11-year sentinel surveillance data providing recent prevalence trends for three sexually transmitted diseases (STDs) among drug users for 2010–2020 in the Guangxi China–Vietnam border area, which is one of the provinces with a high HIV burden in China. The findings of this work are important to understand the epidemic in STDs and to evaluate the measures of HIV prevention and control in Guangxi.

The findings showed that heroin remained the most widely used drug in the China–Vietnam border area in 2020. However, the number of heroin-only users was declining and the proportion of SD-only users and poly-drug users was rising. The same results have been demonstrated previously in studies from Chongqing province [6] and a national estimate [14]. Two reasons could explain this result. On the one hand, opium is the only natural source from which heroin can be purified, and the cultivation of the source of opium (the poppy) requires special land, more time and a higher cost. In contrast, it is easy and low cost to produce synthetic drugs by industrial chemicals that can be purchased legally [14]. Therefore, the lower price and easier availability of synthetic drugs than heroin are the main reasons for their widespread popularity among young people in China. On the other hand, given the rapid growth in the category of new psychoactive substances (NPS) and the lagging nature of synthetic drug laws, some psychotropic drugs are not yet defined as illegal [28], such as betel nut, nitrous oxide and rush popper. However, the Chinese government’s criminal law provides harsher criminal penalties for the sale and use of heroin than for synthetic drugs [13]. This may contribute to the wide prevalence of NPS in the population. Therefore, China needs to quickly improve synthetic drug legislation to control the number of people who use those drugs.

Our study found a decrease in the prevalence of HIV, HCV, and syphilis among heroin-only users and poly-drug users, whereas the prevalence of HCV and HIV among SD-only users was increasing, which is consistent with existing finding [14], suggesting that HIV and HCV infections caused by synthetic drug use should be a cause for alarm and timely measures should be taken control these infections. In addition, the decrease of injected drugs behavior could explain this result. The Chinese government has launched a series of strategies to reduce drug abuse and the harm caused, including a methadone maintenance treatment (MMT) program, free HIV voluntary counseling/testing and a needle exchange [29]. Our study confirmed the effectiveness and benefit of these measures, which was consistent with other studies [6, 30].

There may be three reasons for the rising prevalence of STDs from synthetic drug use. First, methadone is a highly effective alternative to heroin addiction treatment [31], but there are currently no alternatives to synthetic drugs [14] and therefore there are no treatment clinics specifically for synthetic drug users. Second, unlike heroin users, most of the synthetic drug users in this study did not use the drug via injection. This makes the MMT program and needle exchange less effective for SD users. And the proportion of drug users who have ever engaged in commercial sex has increased. This will make it more difficult to control the prevalence of STIs among synthetic drug users. Finally, some synthetic drugs are not subject to existing narcotic drug regulations and people who use these synthetic drugs are not subject to compulsory detoxification [14],which may lead to the widespread prevalence of STDs among synthetic drug users. The government should improve its regulatory policies for synthetic drug users in order to reduce the use of synthetic drugs. Dedicated clinics should be established to help synthetic drug users via a range of measures, including implementing HIV pre-exposure prophylaxis, high-risk behavioral interventions and frequent STDs testing, and also providing counseling and treatment for STDs patients.

This study showed that the largest proportion of SD-only users are 13–35 years old (young people), which is consistent with other related research findings [3, 16, 32]. Part of the reason may be that synthetic drugs have only entered China for little more than 20 years [3, 12, 32]. Synthetic drugs are also known as club drugs because of their association with entertainment venues [15]. Young people at parties may be pressured by peers to try synthetic drugs or to share drugs in their circle of friends [33, 34]. Related research shows that high psychological stress is a major factor in adolescent drug use [33]. Therefore, it is not only necessary to strengthen the awareness of NPS prevention among young people but also to strengthen the mental health education of young people to relieve psychological pressure.

Synthetic drugs have been reported to stimulate sexual desire and prolong sexual intercourse [11, 15, 32]. Previous studies have shown that high-risk sexual behavior was the main factor associated with HIV infection among synthetic drugs users [3, 32], which may indirectly explain why the unmarried/divorced/widowed individuals were more likely to be infected with HIV among the SD-only users in this study. Our study also showed that synthetic drug users were more likely to have sex or commercial sex after using drugs. Related studies have shown that sexual and injecting risk behaviors among drug users increase with age [35, 36], which can explain why older people have a greater chance of contracting STDs than younger people among SD-only users.

For heroin-only users, the risk of HIV and HCV infection was three to five times higher among those who had injected drugs than those who had not. In addition, among the poly-drug users, HIV, HCV and syphilis infections were independently associated with drug injection and needle sharing. According to the World Drug Report, the greatest harm to heroin users is caused by injecting drugs, owing to the risk of HIV or HCV infection through unsafe injection practices [1]. Our study found that poly-drug users had the highest rates of having ever injected drugs and engaged in commercial sex, which undoubtedly increases the risk of STD transmission [5, 19].

Our study found that female drug users were at high risk of contracting syphilis among the three different kinds of drug users. Some female drug addicts might engage in prostitution activities to obtain drug funds, which makes them more likely to contract syphilis. Women are more vulnerable to *Treponema pallidum* than men owing to their special physiological structure and sexual status [37]. Female drug users who engage in commercial sex may also act as a “bridge population” to spread syphilis to the general population. Therefore, it is necessary to promote condom use among female drug users, improve their perception of HIV and HCV risk and reduce high-risk behaviors in order to prevent STD transmission.

### Limitations

There are several limitations to this study. First, it is a cross-sectional study and cannot infer a causal relationship between disease and behavior. Second, our questionnaires involve sensitive issues and rely entirely on self-report, as well as recreational drug use being illegal in China, which may lead to recall and social desirability biases. Third, we were unable to assess whether poly-drug users used heroin first or synthetic drugs first. The sequence of drug use may have an impact on HIV, HCV and syphilis prevalence. Fourth, our analysis did not include sexual behavior information in the multivariate logistic regression which may be significantly associated with HIV, syphilis and HCV. Finally, the RPR or TRUST could be negative after the syphilis treatment, and HIV, syphilis and HCV all have window periods for serological detection where it is possible that a subset of people infected with one or more of these three diseases goes undetected when they are investigated, affecting outcomes.

## Conclusions

We found decreasing trends of HIV, HCV and syphilis prevalence among heroin and poly-drug users, which reflected the effectiveness of the Chinese government’s invention measures for heroin drug users, such as methadone maintenance therapy, clean needle delivery or exchange. However, there were increasing trends of HIV and HCV prevalence among synthetic drug users from 2010 to 2020 in the China–Vietnam border area. These findings emphasize the need for developing timely and comprehensive interventions to reduce the use of synthetic drugs. In addition, a range of measures, including the implementation of HIV pre-exposure prophylaxis, high-risk behavioral interventions, frequent STD testing and counselling and treatment for people with STD, should be implemented to help synthetic drug users and to reduce the transmission of STD.

Figures titles and legends:

### Electronic supplementary material

Below is the link to the electronic supplementary material.

Supplementary Material 1

## Data Availability

The datasets generated and/or analyzed during the current study are not publicly available because of ethical and legal reasons but are available from the corresponding author Hao Liang on reasonable request.

## Declarations

### Statement

All methods were carried out in accordance with relevant guidelines and regulations.
