# Supplementary material for: Trends and associated factors of HIV, HCV and syphilis infection among different drug users in the China–Vietnam border area: an 11-year cross-sectional study (2010–2020)
Source: BMC Infect Dis. 2023 Sep 4;23:575. doi: 10.1186/s12879-023-08239-3 (PMC10478360; doi:10.1186/s12879-023-08239-3)
Supplement: Supplementary file 1 — Supplementary Material 1 [file 12879_2023_8239_MOESM1_ESM.docx]

**Table S1. Prevalence of HIV, HCV and syphilis among the different drug users**

|  | Year, n [%, 95%CI (%)] | | | | | | | | | | | | Chi-square  trend test, *P* |
| --- | --- | --- | --- | --- | --- | --- | --- | --- | --- | --- | --- | --- | --- |
|  | total | 2010 | 2011 | 2012 | 2013 | 2014 | 2015 | 2016 | 2017 | 2018 | 2019 | 2020 |  |
| **HIV** |  |  |  |  |  |  |  |  |  |  |  |  |  |
| Heroin-only | 1787[7.28,  (6.96-7.61)] | 243[12.76,  (11.26-14.26)] | 291[12.44,  (11.10-13.77)] | 221[8.22,  (7.18-9.25)] | 174[6.96,  (5.96-7.96)] | 160[6.22,  (5.29-7.15)] | 111[4.63,  (3.79-5.48)] | 174[11.95,  (10.28-13.62)] | 128[5.85,  (4.86-6.83)] | 114[4.12,  (3.38-4.86)] | 99[4.78,  (3.86-5.69)] | 72[4.35,  (3.36-5.33)] | ＜0.01 |
| Synthetic drug-only | 22[1.07,  (0.62-1.51)] | 1[0.89,  (-0.88-2.66)] | 1[1.39,  (-1.38-4.16)] | 0[0, (0.00-0.00)] | 0[0,  (0.00-0.00)] | 1[0.51,  (-0.50-1.52)] | 3[1.26,  (-0.17-2.68)] | 1[1.08,  (-1.06-3.21)] | 0[0,(0.00-0.00)] | 3[1.14,  (-0.15-2.43)] | 2[1.27,  (-0.50-3.05)] | 10[2.84,  (1.10-4.58)] | ＜0.01 |
| Poly-drug | 163[6.95,  (5.92-7.98)] | 28[18.30,  (12.10-24.50)] | 13[13.98,  (6.80-21.16)] | 18[7.73,  (4.27-11.18)] | 22[10.53,  (6.33-14.72)] | 15[5.62,  (2.84-8.40)] | 19[5.52,  (3.10-7.95)] | 8[3.72,  (1.17-6.27)] | 7[3.08,  (0.82-5.35)] | 8[3.05,  (0.96-5.15)] | 12[7.69,  (3.46-11.92)] | 13[6.95,  (3.27-10.63)] | ＜0.01 |
| Total | 1972[6.81,  (6.52-7.10)] | 272[12.53,  (11.14-13.93)] | 305[12.18,  (10.89-13.46)] | 239[7.89,  (6.93-8.85)] | 196[6.65,  (5.75-7.55)] | 176[5.80,  (4.97-6.63)] | 133[4.47,  (3.72-5.21)] | 183[10.37,  (8.95-11.80)] | 135[5.10,  (4.26-5.93)] | 125[3.80,  (3.15-4.45)] | 113[4.74,  (3.88-5.59)] | 95[4.33,  (3.47-5.18)] | ＜0.01 |
| **HCV** |  |  |  |  |  |  |  |  |  |  |  |  |  |
| Heroin-only | 13,189[53.74,  (53.11-54.36)] | 1150[60.37,  (58.17-62.57)] | 1370[58.55,  (56.55-60.54)] | 1479[54.98,  (53.10-56.86)] | 1274[50.94,  (48.98-52.90)] | 1308[50.86,  (48.92-52.79)] | 1210[50.52,  (48.52-52.53)] | 896[61.54,  (59.04-64.04)] | 1331[60.80,  (58.76-62.85)] | 1310[47.38,  (45.52-49.24)] | 978[47.18,  (45.03-49.33)] | 883[53.29,  (50.88-55.69)] | ＜0.01 |
| Synthetic drug-only | 322[15.62,  (14.05-17.18)] | 10[8.93,  (3.57-14.29)] | 6[8.33,  (1.79-14.87)] | 8[7.41,  (2.39-12.43)] | 4[1.69,  (0.04-3.34)] | 46[23.47,  (17.48-29.45)] | 32[13.39,  (9.04-17.74)] | 15[16.13,  (8.51-23.74)] | 67[28.76,  (22.90-34.61)] | 36[13.69,  (9.51-17.87)] | 32[20.38,  (14.01-26.75)] | 66[18.75,  (14.65-22.85)] | ＜0.01 |
| Poly-drug | 1243[52.98,  (50.96-55.00)] | 102[66.67,  (59.11-74.22)] | 72[77.42,  (68.76-86.08)] | 173[74.25,  (68.59-79.91)] | 145[69.38,  (63.08-75.68)] | 126[47.19,  (41.16-53.22)] | 187[54.36,  (49.07-59.65)] | 99[46.05,  (39.33-52.76)] | 114[50.22,  (43.67-56.77)] | 78[29.77,  (24.20-35.34)] | 95[60.90,  (53.15-68.64)] | 52[27.81,  (21.33-34.29)] | ＜0.01 |
| Total | 14,754[50.96,  (50.39-51.54)] | 1262[58.16,  (56.08-60.23)] | 1448[57.80,  (55.87-59.74)] | 1660[54.77,  (52.99-56.54)] | 1423[48.29, (46.48-50.09)] | 1480[48.76,  (46.99-50.54)] | 1429[47.99,  (46.19-49.78)] | 1010[57.26,  (54.95-59.57)] | 1512[57.08,  [55.19-58.96)] | 1424[43.28,  (41.59-44.98)] | 1105[46.31,  (44.31-48.31)] | 1001[45.58,  (43.50-47.67)] | ＜0.01 |
| **Syphilis** |  |  |  |  |  |  |  |  |  |  |  |  |  |
| Heroin-only | 1274[5.19,  (4.91-5.47)] | 109[5.72,  (4.68-6.77)] | 188[8.03,  (6.93-9.14)] | 167[6.21,  (5.30-7.12)] | 124[4.96,  (4.11-5.81)] | 112[4.35,  (3.57-5.14)] | 122[5.09,  (4.21-5.98)] | 73[5.01,  (3.89-6.14)] | 125[5.71,  (4.74-6.68)] | 114[4.12,  (3.38-4.86)] | 65[3.14,  (2.38-3.89)] | 75[4.53,  (3.52-5.53)] | ＜0.01 |
| Synthetic drug-only | 50[2.42,  (1.76-3.09)] | 1[0.89,  (-0.88-2.66)] | 5[6.94,  (0.93-12.96)] | 2[1.85,  (-0.73-4.44)] | 3[1.27,  (-0.17-2.70)] | 7[3.57,  (0.95-6.19)] | 6[2.51,  (0.51-4.51)] | 3[3.23,  (-0.43-6.88)] | 4[1.72,  (0.04-3.40)] | 5[1.90,  (0.24-3.56)] | 3[1.91,  (-0.25-4.08)] | 11[3.13,  (1.30-4.95)] | 0.885 |
| Poly-drug | 206[8.78,  (7.63-9.93)] | 24[15.69,  (9.86-21.51)] | 11[11.83,  (5.14-18.51)] | 34[14.59,  (10.03-19.16)] | 35[16.75,  (11.64-21.85)] | 14[5.24,  (2.55-7.93)] | 34[9.88,  (6.71-13.05)] | 19[8.84,  (5.01-12.66)] | 7[3.08,  (0.82-5.35)] | 8[3.05,  (0.96-5.15)] | 10[6.41,  (2.52-10.30)] | 10[5.35,  (2.09-8.60)] | ＜0.01 |
| Total | 1530[5.28,  (5.03-5.54)] | 134[6.18,  (5.16-7.19)] | 204[8.14,  (7.07-9.22)] | 203[6.70,  (5.81-7.59)] | 162[5.50,  (4.67-6.32)] | 133[4.38,  (3.65-5.11)] | 162[5.44,  (4.62-6.25)] | 95[5.39,  (4.33-6.44)] | 136[5.13,  (4.29-5.97)] | 127[3.86,  (3.20-4.52)] | 78[3.27,  (2.56-3.98)] | 96[4.37,  (3.52-5.23)] | ＜0.01 |

**Table S2. Trends across the years for proportion of drug type**

|  | Year, n [%, (95%CI)] | | | | | | | | | | | | Chi-square  trend test, *P* |
| --- | --- | --- | --- | --- | --- | --- | --- | --- | --- | --- | --- | --- | --- |
|  | total | 2010 | 2011 | 2012 | 2013 | 2014 | 2015 | 2016 | 2017 | 2018 | 2019 | 2020 |  |
| **Drug type** |  |  |  |  |  |  |  |  |  |  |  |  |  |
| Heroin-only | 24,543[84.77,  (84.36-85.19)] | 1905[87.79,  (86.41-89.17)] | 2340[93.41,  (92.44-94.39)] | 2690[88.75, (87.62-89.88)] | 2501[84.87,  (83.57-86.16)] | 2572[84.74,  (83.46-86.02)] | 2395[80.42,  (79.00-81.85)] | 1456[82.54,  (80.77-84.31)] | 2189[82.63,  (81.19-84.08)] | 2765[84.04,  (82.79-85.29)] | 2073[86.88,  (85.53-88.24)] | 1657[75.46,  (73.65-77.26)] | <0.001 |
| Synthetic drug-only | 2062[7.12,  (6.83-7.42)] | 112[5.16,  (4.23-6.09)] | 72[2.87,  (2.22-3.53)] | 108[3.56,  (2.90-4.22)] | 237[8.04,  (7.06-9.02)] | 196[6.46,  (5.58-7.33)] | 239[8.03,  (7.05-9.00)] | 93[5.27,  (4.23-6.32)] | 233[8.80,  (7.72-9.88)] | 263[7.99,  (7.07-8.92)] | 157[6.58,  (5.58-7.58)] | 352[16.03,  (14.49-17.56)] | <0.001 |
| Poly-drug | 2346[8.10,  (7.79-8.42)] | 153[7.05,  (5.97-8.13)] | 93[3.71,  (2.97-4.45)] | 233[7.69,  (6.74-8.64)] | 209[7.09,  (6.16-8.02)] | 267[8.80,  (7.79-9.81)] | 344[11.55,  (10.40-12.70)] | 215[12.19,  (10.66-13.72)] | 227[8.57,  (7.50-9.64)] | 262[7.96,  (7.04-8.89)] | 156[6.54,  (5.55-7.53)] | 187[8.52,  (7.35-9.68)] | <0.001 |
| Total | 28,951 | 2170 | 2505 | 3031 | 2947 | 3035 | 2978 | 1764 | 2649 | 3290 | 2386 | 2196 | - |

**Table S3. Trends of age group of HIV, HCV and syphilis among drug users**

|  | Year, n (%) | | | | | | | | | | | | Chi-square  trend test, *P* |
| --- | --- | --- | --- | --- | --- | --- | --- | --- | --- | --- | --- | --- | --- |
|  | total | 2010 | 2011 | 2012 | 2013 | 2014 | 2015 | 2016 | 2017 | 2018 | 2019 | 2020 |  |
| **HIV** |  |  |  |  |  |  |  |  |  |  |  |  |  |
| aged 13-34 | 891[45.18, (43.98-47.38)] | 182[66.91, (61.28-72.54)] | 190[62.30, (56.83-67.76)] | 135[56.49, (50.15-62.82)] | 91[46.43, (39.38-53.47)] | 73[41.48, (34.13-48.83)] | 37[27.82, (20.10-35.53)] | 110[60.11, (52.95-67.27)] | 28[20.74, (13.81-27.67)] | 20[16.00, (9.48-22.52)] | 17[15.04, (8.35-21.74)] | 8[8.42, (2.73-14.11)] | ＜0.01 |
| aged ≥35 | 1081[54.82, (52.62-57.02)] | 90[33.09,(27.46-38.72)] | 115[37.70,(32.24-43.17)] | 104[43.51,(37.18-49.85)] | 105[53.57,(46.53-60.62)] | 103[58.52,(51.17-65.87)] | 96[72.18,(64.47-79.90)] | 73[39.89,(32.73-47.05)] | 107[79.26,(72.33-86.19)] | 105[84.00,(77.48-90.52)] | 96[84.96,(78.26-91.65)] | 87[91.58,(85.89-97.27)] | ＜0.01 |
| Total | 1972 | 272 | 305 | 239 | 196 | 176 | 133 | 183 | 135 | 125 | 113 | 95 |  |
| **HCV** |  |  |  |  |  |  |  |  |  |  |  |  |  |
| aged 13-34 | 7387[50.07, (49.26-50.87)] | 825[65.37, (62.74-68.00)] | 902[62.29, (59.79-64.79)] | 1046[63.01, (60.69-65.34)] | 789[55.45, (52.86-58.03)] | 768[51.89, (49.34-54.44)] | 652[45.63, (43.04-48.21)] | 596[59.01, (55.97-62.05)] | 673[44.51, (42.00-47.02)] | 518[36.38, (33.87-38.88)] | 344[31.13, (28.40-33.87)] | 274[27.37, (24.61-30.14)] | ＜0.01 |
| aged ≥35 | 7367[49.93, (49.13-50.74)] | 437(34.63)56(41.79)-)] | 546(37.71)99(48.53)-)] | 614(36.99)111(54.68)-)] | 634(44.55)90(55.56)-)] | 712(48.11)78(58.65)-)] | 777(54.37)110(67.90)-)] | 414(40.99)55(57.89)-)] | 839(55.49)101(74.26)-)] | 906(63.62)103(81.10)-)] | 761(68.87)68(87.18)-)] | 727(72.63)82(85.42)-)] | ＜0.01 |
| Total | 14754 | 1262 | 1448 | 1660 | 1423 | 1480 | 1429 | 1010 | 1512 | 1424 | 1105 | 1001 |  |
| **Syphilis** |  |  |  |  |  |  |  |  |  |  |  |  |  |
| aged 13-34 | 577[37.71, (35.28-40.14)] | 78[58.21, (49.75-66.67)] | 105[51.47, (44.55-58.39)] | 92[45.32, (38.41-52.23)] | 72[44.44, (36.71-52.18)] | 55[41.35, (32.87-49.83)] | 52[32.10, (24.83-39.36)] | 40[42.11, (31.99-52.22)] | 35[25.74, (18.29-33.18)] | 24[18.90, (12.00-25.80)] | 10[12.82, (5.23-20.41)] | 14[14.58, (7.39-21.77)] | ＜0.01 |
| aged ≥35 | 953[62.29, (59.86-64.72)] | 56[41.79,(33.33-50.25)] | 99[48.53,(41.61-55.45)] | 111[54.68,(47.77-61.59)] | 90[55.56,(47.82-63.29)] | 78[58.65,(50.17-67.13)] | 110[67.90,(60.64-75.17)] | 55[57.89,(47.78-68.01)] | 101[74.26,(66.82-81.71)] | 103[81.10,(74.20-88.00)] | 68[87.18,(79.59-94.77)] | 82[85.42,(78.23-92.61)] | ＜0.01 |
| Total | 1530 | 134 | 204 | 203 | 162 | 133 | 162 | 95 | 136 | 127 | 78 | 96 |  |

**Table S4. Trends of behavioral variables among drug users in the China–Vietnam border area of Guangxi from 2010 to 2020, n (%)**

|  | Year, n (%) | | | | | | | | | | | | Chi-square  trend test, *P* |
| --- | --- | --- | --- | --- | --- | --- | --- | --- | --- | --- | --- | --- | --- |
|  | total | 2010 | 2011 | 2012 | 2013 | 2014 | 2015 | 2016 | 2017 | 2018 | 2019 | 2020 |  |
| **Having ever injected drugs** | |  |  |  |  |  |  |  |  |  |  |  |  |
| Yes | 21451[74.09, (73.59-74.60)] | 1722[79.35, (77.65-81.06)] | 1883[75.17, (73.48-76.86)] | 2374[78.32, (76.86-79.79)] | 2163[73.4, (71.80-74.99)] | 2292[75.52, (73.99-77.05)] | 2261[75.92, (74.39-77.46)] | 1373[77.83, (75.89-79.77)] | 1924[72.63, (70.93-74.33)] | 2286[69.48, (67.91-71.06)] | 1648[69.07, (67.21-70.93)] | 1559[70.99, (67.52-71.37)] | ＜0.01 |
| No | 7500[25.91, (25.40-26.41) | 448[20.65, (18.94-22.35)] | 622[24.83, (23.14-26.52)] | 657[21.68, (20.21-23.14)] | 784[26.6, (25.01-28.20)] | 743[24.48, (22.95-26.01)] | 717[24.08, (22.54-25.61)] | 391[22.17, (20.23-24.11)] | 725[27.37, (25.67-29.07)] | 1004[30.52, (28.94-32.09)] | 738[30.93, (29.07-32.79)] | 671[30.56, (28.63-32.48)] | ＜0.01 |
| **Needle sharing** |  |  |  |  |  |  |  |  |  |  |  |  |  |
| Yes | 7357[25.41, (24.91-25.91)] | 598[27.56, (25.68-29.44)] | 528[21.08, (19.48-22.68)] | 784[25.87, (24.31-27.43)] | 696[23.62, (22.08-25.15)] | 669[22.04, (20.57-23.52)] | 854[28.68, (27.05-30.30)] | 690[39.12, (36.84-41.40)] | 873[32.96, (31.16-34.75)] | 699[21.25, (19.85-22.64)] | 329[13.79, (12.40-15.17)] | 637[29.01, (27.11-30.91)] | 0.609 |
| No | 21594[74.59, (74.09-75.09)] | 1977[78.92, (70.56-74.32)] | 2247[74.13, (77.32-80.52)] | 2251[76.38, (72.57-75.69)] | 2366[77.96, (74.85-77.92)] | 2124[71.32, (76.48-79.43)] | 1074[60.88, (69.70-72.95)] | 1776[67.04, (58.60-63.16)] | 2591[78.75, (65.25-68.84)] | 2057[86.21, (77.36-80.15)] | 1559[70.99, (84.83-87.60)] | 1572[72.44, (69.09-72.89)] | 0.609 |
| **Having sex following drug consumption** | | |  |  |  |  |  |  |  |  |  |  |  |
| Yes | 1329[11.48, (10.90-12.06)] | - | - | - | - | - | - | 147[13.88, (11.80-15.97)] | 367[13.85, (12.54-15.17)] | 373[11.34, (10.25-12.42)] | 236[9.89, (8.69-11.09)] | 206[9.38, (8.16-10.60)] | ＜0.01 |
| No | 10251[88.52, (87.94-89.10)] | - | - | - | - | - | - | 912[86.12, (84.03-88.20)] | 2282[86.15, (84.83-87.46)] | 2917[88.66, (87.58-89.75)] | 2150[90.11, (88.91-91.31)] | 1990[90.62, (89.40-91.84)] | ＜0.01 |
| **Having ever engaged in commercial sex** | | | |  |  |  |  |  |  |  |  |  |  |
| Yes | 5827[25.16, (24.60-25.72)] | 513[23.70, (21.90-25.49)] | 438[17.51, (16.02-19.00)] | 658[21.77, (20.30-23.25)] | 543[18.43, (17.02-19.83)] | 569[18.75, (17.36-20.14)] | 612[20.56, (19.11-22.02)] | 376[31.05, (28.44-33.66)] | 719[49.90, (47.31-52.48)] | 553[33.60, (31.31-35.88)] | 524[42.71, (39.93-45.48)] | 322[32.56, (29.63-35.48)] | ＜0.01 |
| No | 17333[74.84, (74.28-75.40) | 1652[76.30, (74.51-78.10)] | 2064[82.49, (81.00-83.98)] | 2364[78.23, (76.75-79.70)] | 2404[81.57, (80.17-82.98)] | 2465[81.25, (79.86-82.64)] | 2364[79.44, (77.98-80.89)] | 835[68.95, (66.34-71.56)] | 722[50.10, (47.52-52.69)] | 1093[66.40, (64.12-68.69)] | 703[57.29, (54.52-60.07)] | 667[67.44, (64.52-70.37)] | ＜0.01 |

"Having sex following drug consumption" was only included in the questionnaire after 2015, and the trend analysis was for the period of 2016-2020.

**Table S5. Crude and adjusted odds ratios for HIV, HCV and syphilis in heroin-only users**

| **Characteristics** | **HIV infection/**  **all participants** | **Crude OR, *P*-value** | **Adjusted OR, *P*-value** | **HCV infection/all participants** | **Crude OR, *P*-value** | **Adjusted OR, *P*-value** | **Syphilis infection/all participants** | **Crude OR, *P*-value** | **Adjusted OR, *P*-value** |
| --- | --- | --- | --- | --- | --- | --- | --- | --- | --- |
| **Gender** |  |  |  |  |  |  |  |  |  |
| Male | 1689/23,746 | Reference. | Reference. | 12762/23,746 | Reference. | Reference. | 1166/23,746 | Reference. | Reference. |
| Female | 98/797 | 1.831(1.474-2.275),0.001 | 1.939(1.535-2.448),0.001 | 427/797 | 0.993(0.862-1.144),0.926 |  | 108/797 | 3.035(2.458-3.749),0.001 | 3.267(2.631-4.056),0.001 |
| **Age** |  |  |  |  |  |  |  |  |  |
| Aged 13-34 | 816/13,086 | Reference. | Reference. | 6665/13,086 | Reference. | Reference. | 486/13,086 | Reference. | Reference. |
| Aged ≥35 | 971/11,457 | 1.392(1.264-1.534),0.001 | 1.651(1.484-1.837),0.001 | 6524/11,457 | 1.274(1.211-1.340),0.001 | 1.322(1.247-1.402),0.001 | 788/11,457 | 1.915(1.705-2.150),0.001 | 2.211(1.955-2.502),0.001 |
| **Marriage status** |  |  |  |  |  |  |  |  |  |
| Married/Cohabitated | 614/9644 | Reference. | Reference. | 5045/9644 | Reference. | Reference. | 555/9644 | Reference. | Reference. |
| Unmarried/Divorced/Widowed | 1173/14,899 | 1.257(1.136-1.391),0.001 | 1.436(1.287-1.601),0.001 | 8144/148,99 | 1.099(1.044-1.157),0.001 | 1.100(1.038-1.166),0.001 | 719/14,899 | 0.830(0.741-0.930),0.001 | 1.036(0.919-1.168),0.559 |
| **Education** |  |  |  |  |  |  |  |  |  |
| <9 years | 1708/23,040 | Reference. | Reference. | 12287/23,040 | Reference. | Reference. | 1183/23,040 | Reference. | Reference. |
| ≥9 years | 79/1503 | 0.693(0.550-0.874),0.002 | 0.770(0.606-0.979),0.033 | 902/1503 | 1.313(1.181-1.461),0.001 | 1.434(1.278-1.609),0.001 | 91/1503 | 1.191(0.956-1.484),0.120 | 1.163(0.931-1.453),0.183 |
| **Ethnicity** |  |  |  |  |  |  |  |  |  |
| Han | 987/10,831 | Reference. | Reference. | 6596/10,831 | Reference. | Reference. | 627/10,831 | Reference. | Reference. |
| Other | 800/13,712 | 0.618(0.561-0.681),0.001 | 0.713(0.641-0.793),0.001 | 6593/13,712 | 0.595(0.565-0.626),0.001 | 0.747(0.704-0.792),0.001 | 647/13,712 | 0.806(0.720-0.902),0.001 | 0.886(0.785-1.000),0.050 |
| **Awareness of HIV-related knowledge** | |  |  |  |  |  |  |  |  |
| No | 115/1333 | Reference. | Reference. | 750/1333 | Reference. | Reference. | 92/1333 | Reference. | Reference. |
| Yes | 1672/23,210 | 0.822(0.675-1.002),0.052 | 0.960(0.778-1.183),0.700 | 12439/23,210 | 0.898(0.803-1.003),0.057 | 1.036(0.914-1.173),0.580 | 1182/23,210 | 0.724(0.581-0.902),0.004 | 0.797(0.636-0.999),0.049 |
| **Having ever injected drugs** |  |  |  |  |  |  |  |  |  |
| NO | 60/5361 | Reference. | Reference. | 1108/5361 | Reference. | Reference. | 226/5361 | Reference. | Reference. |
| Yes | 1727/19,182 | 8.741(6.745-11.328),0.001 | 3.796(2.900-4.968),0.001 | 12081/19,182 | 6.530(6.075-7.020),0.001 | 5.019(4.648-5.421),0.001 | 1048/19,182 | 1.313(1.134-1.521),0.001 | 1.216(1.038-1.425),0.015 |
| **Needle sharing** |  |  |  |  |  |  |  |  |  |
| No | 650/17,916 | Reference. | Reference. | 8219/17,916 | Reference. | Reference. | 887/17,916 | Reference. | Reference. |
| Yes | 1137/6627 | 5.501(4.973-6.086),0.001 | 4.157(3.735-4.627),0.001 | 4970/6627 | 3.839(3.323-3.768),0.001 | 2.290(2.141-2.449),0.001 | 387/6627 | 1.191(1.053-1.346),0.005 | 1.072(0.939-1.223),0.302 |
| **Having ever received free condom or HIV testing and counseling services** | | |  |  |  |  |  |  |  |
| No | 433/4397 | Reference. | Reference. | 2731/4397 | Reference. | Reference. | 281/4397 | Reference. | Reference. |
| Yes | 1354/20,146 | 0.660(0.589-0.739),0.001 | 0.814(0.714-0.929),0.002 | 10458/20,146 | 0.659(0.616-0.704),0.001 | 0.867(0.800-0.938),0.001 | 993/20,146 | 0.759(0.662-0.871),0.001 | 0.890(0.764-1.037),0.136 |
| **Having ever received free methadone maintenance therapy or clean needles** | | | |  |  |  |  |  |  |
| No | 537/8163 | Reference. | Reference. | 4668/9163 | Reference. | Reference. | 496/8163 | Reference. | Reference. |
| Yes | 1250/16,380 | 1.173(1.057-1.303),0.001 | 1.198(1.047-1.370),0.009 | 8521/16,380 | 0.812(0.769-0.856),0.001 | 0.724(0.674-0.778),0.001 | 778/16,380 | 0.771(0.686-0.866),0.001 | 0.748(0.646-0.865),0.001 |
| **Having ever received peer education services** | | |  |  |  |  |  |  |  |
| No | 875/12,873 | Reference. | Reference. | 7193/128,73 | Reference. | Reference. | 701/12,873 | Reference. | Reference. |
| Yes | 912/11,670 | 1.162(1.056-1.280),0.002 | 1.135(1.010-1.276),0.033 | 5996/11,670 | 0.834(0.794-0.877),0.001 | 1.015(0.953-1.082),0.640 | 573/11,670 | 0.897(0.801-1.004),0.059 | 1.045(0.913-1.196),0.521 |
| **Sampled Year** |  |  |  |  |  |  |  |  |  |
| 2010-2013 | 929/9436 | Reference. | Reference. | 5273/9436 | Reference. | Reference. | 588/9436 | Reference. | Reference. |
| 2014-2017 | 573/8612 | 0.653(0.586-0.727),0.001 | 0.500(0.446-0.561),0.001 | 4745/8612 | 0.969(0.913-1.027),0.290 | 0.874(0.819-0.932),0.001 | 432/8612 | 0.795(0.699-0.903),0.001 | 0.735(0.645-0.838),0.001 |
| 2018-2020 | 285/6495 | 0.420(0.367-0.482),0.001 | 0.378(0.327-0.438),0.001 | 3171/6495 | 0.753(0.707-0.802),0.001 | 0.776(0.722-0.834),0.001 | 254/6495 | 0.612(0.527-0.712),0.001 | 0.530(0.453-0.620),0.001 |

**Table S6. Crude and adjusted odds ratios for HIV, HCV and syphilis in SD-only users**

| **Characteristics** | **HIV infection/all participants** | **Crude OR,** P**-value** | **Adjusted OR,** P**-value** | **HCV infection/all participants** | **Crude OR,** P**-value** | **Adjusted OR, *P*-value** | **Syphilis infection/all participants** | **Crude OR,** P**-value** | **Adjusted OR,** P**-value** |
| --- | --- | --- | --- | --- | --- | --- | --- | --- | --- |
| **Gender** |  |  |  |  |  |  |  |  |  |
| Male | 21/1895 | Reference. | Reference. | 310/1895 | Reference. | Reference. | 42/1895 | Reference. | Reference. |
| Female | 1/167 | 0.538(0.072-4.022),0.545 |  | 12/167 | 0.396(0.217-0.721),0.002 | 0.637(0.340-1.193),0.159 | 8/167 | 2.220(1.024-4.810),0.043 | 2.822(1.272-6.260),0.011 |
| **Age** |  |  |  |  |  |  |  |  |  |
| Aged 13-34 | 9/1442 | Reference. | Reference. | 156/1442 | Reference. | Reference. | 25/1442 | Reference. | Reference. |
| Aged ≥35 | 13/620 | 3.410(1.450-8.020),0.005 | 2.820(1.083-7.342),0.034 | 166/620 | 3.014(2.363-3.845),0.001 | 2.186(1.682-2.841),0.001 | 25/620 | 2.382(1.357-4.180),0.003 | 2.603(1.463-4.631),0.001 |
| **Marriage status** |  |  |  |  |  |  |  |  |  |
| Married/Cohabitated | 4/778 | Reference. | Reference. | 124/778 | Reference. | Reference. | 18/778 | Reference. | Reference. |
| Unmarried\Divorced/Widowed | 18/1284 | 2.751(0.928-8.159),0.068 | 4.419(1.433-13.629),0.010 | 198/1284 | 0.962(0.753-1.228),0.754 |  | 31/1284 | 0.988(0.554-1.762),0.968 |  |
| **Education** |  |  |  |  |  |  |  |  |  |
| <9 years | 20/1820 | Reference. | Reference. | 298/1820 | Reference. | Reference. | 46/1820 | Reference. | Reference. |
| ≥9 years | 2/242 | 0.750(0.174-3.229),0.669 |  | 24/242 | 0.562(0.362-0.873),0.010 | 0.636(0.398-1.016)0.058 | 4/242 | 0.648(0.231-1.817),0.410 |  |
| **Ethnicity** |  |  |  |  |  |  |  |  |  |
| Han | 9/979 | Reference. | Reference. | 201/979 | Reference. | Reference. | 29/979 | Reference. | - |
| Other | 13/1083 | 1.309(0.557-3.077),0.536 |  | 121/1083 | 0.487(0.381-0.622),0.001 | 0.525(0.396-0.696),0.001 | 21/1083 | 0.648(0.367-1.144),0.134 | 0.657(0.370-1.166),0.151 |
| **Awareness of HIV-related knowledge** | | |  |  |  |  |  |  |  |
| No | 2/119 | Reference. | Reference. | 23/119 | Reference. | Reference. | 2/119 | Reference. | Reference. |
| Yes | 20/1943 | 0.609(0.141-2.634),0.506 |  | 299/1943 | 0.759(0.474-1.216),0.252 |  | 48/1943 | 1.482(0.356-6.172),0.589 |  |
| **Having ever injected drugs** |  |  |  |  |  |  |  |  |  |
| NO | 11/1738 | Reference. | Reference. | 206/1738 | Reference. | Reference. | 42/1738 | Reference. | Reference. |
| Yes | 11/324 | 5.518(2.372-12.837),0.001 | 2.161(0.690-6.768),0.186 | 116/324 | 4.147(3.167-5.431),0.001 | 3.696(2.604-5.247),0.001 | 8/324 | 1.022(0.475-2.198),0.955 |  |
| **Needle sharing** |  |  |  |  |  |  |  |  |  |
| No | 17/1964 | Reference. | Reference. | 290/1964 | Reference. | Reference. | 47/1964 | Reference. | Reference. |
| Yes | 5/98 | 6.157(2.223-17.052),0.001 | 1.704(0.477-6.089),0.412 | 32/98 | 2.799(1.802-4.347),0.001 | 0.779(0.459-1.322),0.355 | 3/98 | 1.288(0.394-4.213),0.676 |  |
| **Having ever received free condom or HIV testing and counseling services** | | | |  |  |  |  |  |  |
| No | 4/672 | Reference. | Reference. | 39/672 | Reference. | Reference. | 16/672 | Reference. | Reference. |
| Yes | 18/1390 | 2.191(0.739-6.499),0.157 | 0.889(0.266-2.964),0.848 | 233/1390 | 1.319(1.013-1.717),0.040 | 0.974(0.716-1.325),0.868 | 34/1390 | 1.028(0.563-1.876),0.928 |  |
| **Having ever received free methadone maintenance therapy or clean needles** | | | |  |  |  |  |  |  |
| No | 11/1701 | Reference. | Reference. | 225/1701 | Reference. | Reference. | 39/1701 | Reference. | Reference. |
| Yes | 11/361 | 4.829(2.077-11.225),0.001 | 1.927(0.637-5.831),0.246 | 97/361 | 2.410(1.837-3.163),0.001 | 1.649(1.158-2.350),0.006 | 11/361 | 1.339(0.679-2.641),0.399 |  |
| **Having ever received peer education services** | | |  |  |  |  |  |  |  |
| No | 13/1713 | Reference. | Reference. | 251/1713 | Reference. | Reference. | 37/1713 | Reference. | Reference. |
| Yes | 9/349 | 3.462(1.468-8.162),0.005 | 1.834(0.611-5.504),0.280 | 71/349 | 1.488(1.110-1.994),0.008 | 0.789(0.544-1.145),0.213 | 13/349 | 1.753(0.922-3.333),0.087 | 1.715(0.898-3.277),0.103 |
| **Sampled Year** |  |  |  |  |  |  |  |  |  |
| 2010-2013 | 2/529 | Reference. | Reference. | 28/529 | Reference. | Reference. | 11/529 | Reference. | Reference. |
| 2014-2017 | 5/761 | 1.743(0.337-0.9016),0.508 | 1.057(0.191-5.859),0.950 | 160/761 | 4.763(3.134-7.240),0.001 | 3.278(2.083-5.157),0.001 | 20/761 | 1.271(0.604-2.675),0.528 |  |
| 2018-2020 | 15/772 | 5.221(1.189-22.927),0.029 | 2.659(0.549-12.893),0.225 | 134/772 | 3.758(2.460-5.741),0.001 | 2.174(1.369-3.455),0.001 | 19/772 | 1.188(0.561-2.518),0.653 |  |

**Table S7. Crude and adjusted odds ratios for HIV, HCV and syphilis in poly-drug users**

| **Characteristics** | **HIV infection/all participants** | **Crude OR, *P*-value** | **Adjusted OR, *P*-value** | **HCV infection/all participants** | **Crude OR, *P*-value** | **Adjusted OR, *P*-value** | **Syphilis infection/all participants** | **Crude OR, *P*-value** | **Adjusted OR, *P*-value** |
| --- | --- | --- | --- | --- | --- | --- | --- | --- | --- |
| **Gender** |  |  |  |  |  |  |  |  |  |
| Male | 157/2252 | Reference. | Reference. | 1198/2252 | Reference. | Reference. | 188/2252 | Reference. | Reference. |
| Female | 6/94 | 0.910(0.392-2.113),0.826 |  | 45/94 | 0.808(0.535-1.221),0.312 |  | 18/94 | 2.600(1.523-4.440),0.001 | 2.806(1.592-4.946),0.001 |
| **Age** |  |  |  |  |  |  |  |  |  |
| Aged 13-34 | 66/1132 | Reference. | Reference. | 566/1132 | Reference. | Reference. | 66/1132 | Reference. | Reference. |
| Aged ≥35 | 97/1214 | 1.403(1.015-1.939),0.041 | 1.587(1.122-2.246),0.009 | 677/1214 | 1.261(1.072-1.483),0.005 | 1.442(1.200-1.733),0.001 | 140/1214 | 2.105(1.552-2.855),0.001 | 2.515(1.815-3.486),0.001 |
| **Marriage status** |  |  |  |  |  |  |  |  |  |
| Married/Cohabitated | 44/797 | Reference. | Reference. | 419/797 | Reference. | Reference. | 80/797 | Reference. | Reference. |
| Unmarried\Divorced/Widowed | 119/1549 | 1.424(0.997-2.035),0.052 | 1.360(0.937-1.973),0.106 | 824/1549 | 1.025(0.864-1.217),0.774 |  | 126/1549 | 0.794(0.591-1.065),0.124 | 0.986(0.721-1.347),0.928 |
| **Education** |  |  |  |  |  |  |  |  |  |
| <9 years | 152/2121 | Reference. | Reference. | 1101/2121 | Reference. | Reference. | 171/2121 | Reference. | Reference. |
| ≥9 years | 11/225 | 0.666(0.355-1.248),0.204 |  | 142/225 | 1.585(1.193-2.105),0.001 | 1.511(1.109-2.059),0.009 | 35/225 | 2.101(1.418-3.112),0.001 | 1.927(1.273-2.919),0.002 |
| **Ethnicity** |  |  |  |  |  |  |  |  |  |
| Han | 78/1124 | Reference. | Reference. | 581/1124 | Reference. | Reference. | 89/1124 | Reference. | Reference. |
| Other | 85/1222 | 1.003(0.729-1.379),0.988 |  | 662/1222 | 1.105(0.939-1.300),0.229 |  | 117/1222 | 1.231(0.923-1.643),0.157 | 0.861(0.633-1.170),0.339 |
| **Awareness of HIV-related knowledge** | | |  |  |  |  |  |  |  |
| No | 15/173 | Reference. | Reference. | 87/173 | Reference. | Reference. | 21/173 | Reference. | Reference. |
| Yes | 148/2173 | 0.770(0.442-1.341),0.356 |  | 1156/2173 | 1.124(0.824-1.532),0.461 |  | 185/2173 | 0.674(0.417-1.089),0.107 | 0.627(0.381-1.034),0.067 |
| **Having ever injected drugs** |  |  |  |  |  |  |  |  |  |
| No | 3/401 | Reference. | Reference. | 103/401 | Reference. | Reference. | 14/401 | Reference. | Reference. |
| Yes | 160/1945 | 11.892(3.776-37.455),0.001 | 6.318(1.955-20.413),0.002 | 1140/1945 | 4.097(3.218-5.216),0.001 | 4.004(3.022-5.305),0.001 | 192/1945 | 3.028(1.740-5.267),0.001 | 2.090(1.159-3.770),0.014 |
| **Needle sharing** |  |  |  |  |  |  |  |  |  |
| No | 73/1714 | Reference. | Reference. | 778/1714 | Reference. | Reference. | 133/1714 | Reference. | Reference. |
| Yes | 90/632 | 3.733(2.701-5.159),0.001 | 2.708(1.938-3.782),0.001 | 465/632 | 3.350(2.741-4.095),0.001 | 2.507(2.017-3.115),0.001 | 73/632 | 1.552(1.148-2.098),0.004 | 1.278(0.927-1.761),0.135 |
| **Having ever received free condom or HIV testing and counseling services** | | | |  |  |  |  |  |  |
| No | 54/586 | Reference. | Reference. | 355/586 | Reference. | Reference. | 56/586 | Reference. | Reference. |
| Yes | 109/1760 | 0.650(0.463-0.914),0.013 | 0.670(0.454-0.988),0.044 | 888/1760 | 0.663(0.548-0.801),0.001 | 0.711(0.563-0.898),0.004 | 150/1760 | 0.882(0.639-1.217),0.444 |  |
| **Having ever received free methadone maintenance therapy or clean needles** | | | | |  |  |  |  |  |
| No | 45/788 | Reference. | Reference. | 442/788 | Reference. | Reference. | 55/788 | Reference. | Reference. |
| Yes | 118/1558 | 1.353(0.949-1.929),0.095 | 1.138(0.750-1.727),0.543 | 801/1558 | 0.828(0.697-0.984),0.032 | 0.488(0.388-0.615),0.001 | 151/1558 | 1.430(1.037-1.972),0.029 | 1.004(0.690-1.462),0.982 |
| **Having ever received peer education services** | | | |  |  |  |  |  |  |
| No | 80/1272 | Reference. | Reference. | 641/1272 | Reference. | Reference. | 93/1272 | Reference. | Reference. |
| Yes | 83/1074 | 1.248(0.908-1.716),0.173 | 1.316(0.907-1.909),0.148 | 602/1074 | 1.256(1.067-1.478),0.006 | 1.387(1.133-1.697),0.002 | 113/1074 | 1.491(1.118-1.987),0.006 | 1.391(0.995-1.945),0.053 |
| **Sampled Year** |  |  |  |  |  |  |  |  |  |
| 2010-2013 | 81/688 | Reference. | Reference. | 492/688 | Reference. | Reference. | 104/688 | Reference. | Reference. |
| 2014-2017 | 49/1053 | 0.366(0.253-0.529),0.001 | 0.379(0.254-0.568),0.001 | 526/1053 | 0.398(0.324-0.488),0.001 | 0.412(0.329-0.516),0.001 | 74/1053 | 0.424(0.310-0.582),0.001 | 0.339(0.241-0.478),0.001 |
| 2018-2020 | 33/605 | 0.432(0.284-0.658),0.001 | 0.496(0.316-0.778),0.002 | 225/605 | 0.236(0.187-0.298),0.001 | 0.252(0.195-0.326),0.001 | 28/605 | 0.272(0.177-0.420),0.001 | 0.247(0.157-0.390),0.001 |
